# Consequences of the coronavirus pandemic for global health research and practice

**DOI:** 10.7189/jogh.10.010366

**Published:** 2020-06

**Authors:** Alexander Supady

**Affiliations:** 1 Department of Medicine III (Interdisciplinary Medical Intensive Care), Medical Center, Faculty of Medicine, University of Freiburg, Germany; 2 Department of Cardiology and Angiology I, Heart Center, University of Freiburg, Germany; 3 Heidelberg Institute of Global Health, University of Heidelberg, Germany

In December 2019, a series of unexplained cases of pneumonia has come to light in the city of Wuhan in China. In virologic analyses of samples from the patients' deep respiratory tract, a novel coronavirus was isolated (first named 2019-nCoV, then SARS-CoV-2). The disease spread rapidly in the city of Wuhan in early 2020 and soon far beyond [1]. On 30 January 2020, the Director-General of the World Health Organization (WHO) declared the outbreak a public health emergency of international concern, and on 11 March 2020, the WHO declared the virus a pandemic [2].

During the past weeks, a rapidly growing body of scientific evidence has been published. Researchers and clinicians from the epicenters of the disease outbreak, particularly in China and in Italy, presented clinical and scientific evidence and reported experiences from the treatment of this novel disease that will help health professionals, researchers and responsible persons in public health in countries, which still prepare for seriously rising numbers of infected patients.

The discussion primarily focuses on clinical treatments and public health responses in high- and middle-income countries. But, due to the pandemic spread of the virus, we must expect further spreading of the disease to countries with weak public health and health care systems, which may easily be overwhelmed by the occurrence of many patients within a short period.

We must consider the potential impact of current travel and movement restrictions on global health interventions and research projects. By nature, global health practice and research heavily relies on international exchange and free international movement of persons and goods. Travel restrictions, if upheld for a longer time, may interfere with project plans and interventions. Vulnerable individuals and populations may suffer from interruptions in the provision of health services. Ongoing projects and interventions may be paused, planned projects may be postponed – this can affect the lives and the well-being of many people.

The risk management of the COVID-19-pandemic in the context of global health research and practice not only needs to take into account microbiological and epidemiological knowledge and expertise on the characteristics and spread of SARS-CoV-2, but also social and economic impacts and challenges in different countries and settings. On the one hand, the resumption of temporarily interrupted projects may endanger people by unintended transmission of the virus from researchers, aid workers and other project staff to people in vulnerable settings. Nearly all populations within the focus of global health research or interventions must be considered at a particular risk. Clinical data suggest that elderly and immunocompromised persons are at a very high risk of infections and poor outcomes when infected with SARS-CoV-2, the high contagiousness of the virus puts people in densely populated settings, such as refugee camps, at a particular risk [3].

On the other hand, disease control measures successfully implemented in high-income countries, may not be the preferred nor the adequate solution for all countries and settings. Sustained lockdowns, first imposed on the city of Wuhan in China and later announced in almost all countries seriously affected by the pandemic, may not be rational everywhere, considering practicability, proportionality and potential side-effects.

Proportional and context-adequate measures need to reflect the age-structure of the populations and further risk-factors for developing serious or fatal courses of COVID-19. Young people are at a lower risk for developing serious COVID-19 than elderly, whereas non-communicable diseases such as hypertension, diabetes, pulmonary or heart disease, that are prevalent in many low- and middle-income countries, are known risk factors for unfavorable courses of COVID-19 [4,5]. Therefore, information on the age-structures and diseases and risk-factors in different communities and societies need to be included critically in the decision-making and project-planning process.

Equally important, though, are the economic and social implications of the interventions. In many resource-constrained settings, people work as day-laborers or they live from subsistence economy; many live in densely populated urban settings. Under these conditions, sustained lockdowns, movement and contact restrictions are not only impossible to upheld, but they will also have serious side-effects such as further impoverishment and hunger, which also endanger the health and survival of the people.

Project managers and people in charge must very cautiously discuss and evaluate the balance of risks of extended interruption and continuation before resuming the projects. This evaluation process may yield different results and consequences for different settings.

**Figure:**
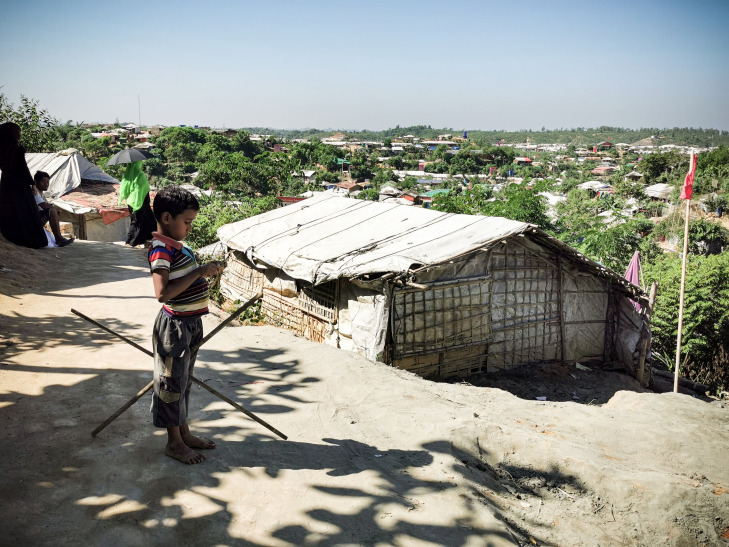
Photo: Kutupalong Rohingya Refugee Camp in Bangladesh. Densely populated refugee camps are highly vulnerable to the spread of coronavirus (from the author’s own collection, used with permission).

The more we learn about the spread and contagiousness of SARS-CoV-2 the better we can develop strategies to resume project work while providing safety to people at risk. The availability and the use of personal protective equipment (PPE) and the possibility to wash or sterilize hands regularly need to become routine in global health projects.

The coronavirus pandemic may thus help to reconsider and redesign the basic structure and foundations of global health research and practice. Recently, the contradiction between frequent international air travel of global health professionals and researchers and efforts of mitigation of climate change has been discussed [6]. This discussion may well be extended to questions of disease prevention in light of international spread of pathogens. Further and more extensive strengthening of local capacities and empowerment of human resources in many low- and middle-income countries that are in the focus of global health activities will help to decrease the demand of deployment of foreign staff. Long distance air travel for short meetings should be reduced. Instead, long term exchanges of staff can strengthen institutional ties and help to guarantee consistency in the projects. Technology may also play an increasing role in global health projects – video calls and conferences should be used more often in project planning and team exchange. Therefore, strategic planning in international cooperation and global health should also keep an eye on the spread of digital technologies and the availability of broad-band internet connections – mobile or landline network based.

The ongoing coronavirus pandemic puts particularly strains on the global health system. We may use this exceptional situation to reconsider and restructure the system for the better of global population health and well-being.
